# Metric and fault-tolerant metric dimension for GeSbTe superlattice chemical structure

**DOI:** 10.1371/journal.pone.0290411

**Published:** 2023-11-30

**Authors:** Liu Liqin, Khurram Shahzad, Abdul Rauf, Fairouz Tchier, Adnan Aslam

**Affiliations:** 1 College of Big Data and Artificial, Anhui Xinhua University, Hefei, Anhui, China; 2 Department of Mathematics, Air University Islamabad, Multan Campus, Multan, Pakistan; 3 Mathematics Department, College of Science, King Saud University, Riyadh, Saudi Arabia; 4 Department of Natural Sciences and Humanities, University of Engineering and Technology, Lahore RCET, Pakistan; China University of Geosciences, CHINA

## Abstract

The concept of metric dimension has many applications, including optimizing sensor placement in networks and identifying influential persons in social networks, which aids in effective resource allocation and focused interventions; finding the source of a spread in an arrangement; canonically labeling graphs; and inserting typical information in low-dimensional Euclidean spaces. In a graph *G*, the set *S*⊆*V*(*G*) of minimum vertices from which all other verticescan be uniquely determined by the distances to the vertices in *S* is called the resolving set. The cardinality of the resolving set is called the metric dimension. The set *S* is called fault-tolerant resolving set if *S*\{*v*} is still a resolving set of *G*. The minimum cardinality of such a set *S* is called fault-tolerant metric dimension of *G*. *GeSbTe* super lattice is the latest chemical compound to have electronic material that is capable of non-volatile storing phase change memories with minimum energy usage. In this work, we calculate the resolving set (fault tolerant resolving set) to find the metric dimension(fault-tolerant metric dimension) for the molecular structure of the *GeSbTe* lattice. The results may be useful in comparing network structure and categorizing the structure of the *GeSbTe* lattice.

## Introduction

Let *G* be a simple, connected graph with vertex and edge set denoted by *V*(*G*) and *E*(*G*) respectively. We use the notations *α* and *β* to denote the order and size of *G*. The distance between two vertices *a*, *b*∈*V*(*G*), denoted by *d*(*a*, *b*) is the length of the shortest path between them. Let $W=\{a_{1},a_{2},...,a_{m}\}\subset V(G)$ be an ordered set. Let *a*∈*V*(*G*) and $r(a,W)=(d(a,a_{1}),d(a,a_{2}),...,d(a,a_{m}))$ be the representation of *a* with respect to *W* as the *m*-tuple. The set *W* is said to be a resolving set if r(a,W)≠r(b,W) for any two distinct vertices *a* and *b* in *V*(*G*)\*W*. The minimum cardinality of resolving set is called metric dimension of *G*, denoted by *dim*(*G*).

The idea of resolving set was first introduced by Slater [1] in 1975. After that Harary and Melter [2] suggested the similar concept and named it metric dimension. Chartrand et al [3] proposed the idea of metric bases and the cardinality of metric bases is referred as metric dimension. After these papers, a lot of work is done in this direction with applications in many fields including technology, Sciences and Social Sciences. The applications of metric dimension appears in numerous scientific zones, such as the route of robots in mechanical autonomy [4], deciding steering conventions topographically, and telecommunication [5]. Some applications of resolving set in chemistry was discussed by Chartrand et al [3].

The answer to the question whether the metric dimension of a graph is a finite number was given by Caceres in [6]. They proved that for any integer *k*≥0 there exist an infinite graph with metric dimension *k* and this number is infinite for infinite comb graph. The computational difficulty of metric dimension in terms of other graph parameters was explored by Gary and Johnson [7]. The metric dimension of Cayley digraphs and Cayley graphs were studied in [3, 8] respectively. Vertik and Ahmad [9] computed the metric dimension of categorical product of graphs [9]. The readers can see [3, 10–12] for more details on metric dimension of graph.

The resolving set *W* of a graph *G* is called fault-tolerant if for every *a*∈*W*, the set *W*\{*a*} is also a resolving set for *G*. The minimum cardinality of this set is called fault-tolerant metric dimension and its elements are called metric bases of *G*.

The fault-tolerant metric dimension of certain crystal structures was determined by Krishnan and Rajan [13]. Raza et al. computed the fault-tolerant metric dimension of certain rotationally symmetric convex polytopes in [14, 15]. Nadeem and Azeem [16] calculated the metric dimension of Hexagonal mobius ladder. The research [17] focuses on computing the fault-tolerant metric dimension for certain network topologies (triangular snake, ladder, Mobius ladder, and hexagonal ladder networks) and finds that the fault-tolerant metric dimension and metric dimension differ by one in all of these network classes. The study [18] discusses fault-tolerant designs for pyramid, OTIS, bicapped, and mesh-derived networks utilizing interconnection networks $P_{k}^{j}$ and $C_{k}^{j}$ graphs, allowing for stable operation even in the face of faults. For more results on fault-tolerant metric dimension of different graphs, see [19–21].

Metric dimension and fault-tolerant metric dimension, among other things, have potential uses in telephony, robot navigation, and geographical routing protocols [22]. In computer networks, metric dimension can be used to determine the minimum number of sensors or monitoring nodes required to observe and diagnose the behavior of the network. By selecting a set of nodes with the smallest metric dimension, we can efficiently monitor the network’s performance and detect faults or attacks. In sensor networks or Internet of Things (IoT) applications, metric dimension plays a role in optimizing the placement of sensors. By strategically selecting sensor locations with high metric dimension, we can ensure effective coverage of the monitored area while minimizing the number of sensors required [23]. Metric dimension can be utilized in route planning and navigation algorithms. By constructing a graph with vertices representing locations and edges representing distances between them, the metric dimension can help identify the minimum set of landmarks or waypoints necessary for efficient route calculation. Metric dimension can be employed in clustering algorithms to determine representative points or prototypes that best capture the structure and characteristics of a dataset. By selecting a minimal set of points with high metric dimension, we can effectively summarize the data and facilitate efficient clustering [24].

The computation of fault-tolerant metric dimension of a graph is a difficult problem and has applications in censor networks. If we consider the vertices in a resolving set as the positions for loran/sonar stations, the location of each vertex can be distinctly determined by its vertex distances from the station site. From this viewpoint, a fault-tolerant (unique) resolving set can be defined as one that still maintains the property of a resolving set even when excluding a station at a uniquely determined vertex location within the resolving set. As a result, fault-tolerant resolving sets expand the usefulness of conventional resolving sets in graphs. Moreover, this demonstrates that the fault-tolerant metric dimension offers a more advantageous practicality compared to the metric dimension [20, 25–28].

In this article, we have computed the metric and fault metric dimension for GeSbTe (Germanium Antimony Telluride) Superlattice. The impetus for researching the Metric and Fault-tolerant Metric Dimensions of GeSbTe (Germanium Antimony Telluride) Superlattice stems from materials science and nanotechnology, namely the design and optimization of phase-change materials used in nonvolatile memory systems. These dimensions reveal structural features of phase-change materials, which are critical in nonvolatile memory systems. Researchers can optimize material design by analyzing these dimensions, resulting in more efficient and stable memory systems.

## GeSbTe super lattice

*GeSbTe* (Germanium Antimony Telluride) superlattice is a material system that has garnered significant attention in the field of phase-change memory and other related applications. It consists of alternating layers of different compounds, namely Germanium Telluride (*GeTe*) and Antimony Telluride (*Sb*_2_*Te*_3_), forming a periodic structure known as a superlattice.

One of the notable properties of *GeSbTe* superlattice is its ability to undergo rapid and reversible phase transitions between amorphous and crystalline states when subjected to certain stimuli such as heat or electrical pulses. This Phase change memory (PCM), an emerging method for nonvolatile information storage, offers a powerful combination of speed and density, both of which are crucial in the age of big data [29–31]. On the other hand, PCM is an excellent choice for wide range of complex application including thermal emitters [32], flexible screens [33]. Although *Ge*_2_*Sb*_2_*Te*_5_ alloys is the most advanced PCM material [34, 35] but still it’s REEST power consumption is high [34, 35]. It has been observed that the power consumption of the PCM material known as *GeSbTe* superlattice is very low [36]. A lot of research has been done to explore the approaches to achieve the *GeSbTe* supper lattice transition [37, 38].

*GeSbTe* superlattice has several advantages as a phase-change material. It exhibits fast switching speeds, high endurance, and good scalability, which are crucial factors for memory applications. Additionally, it demonstrates good thermal stability, allowing reliable operation over a wide range of temperatures. The unique combination of these properties has made GeSbTe superlattice a promising candidate for next-generation non-volatile memory technologies.

## Metric dimension of *GeSbTe* superlattice

**Fig 1:**
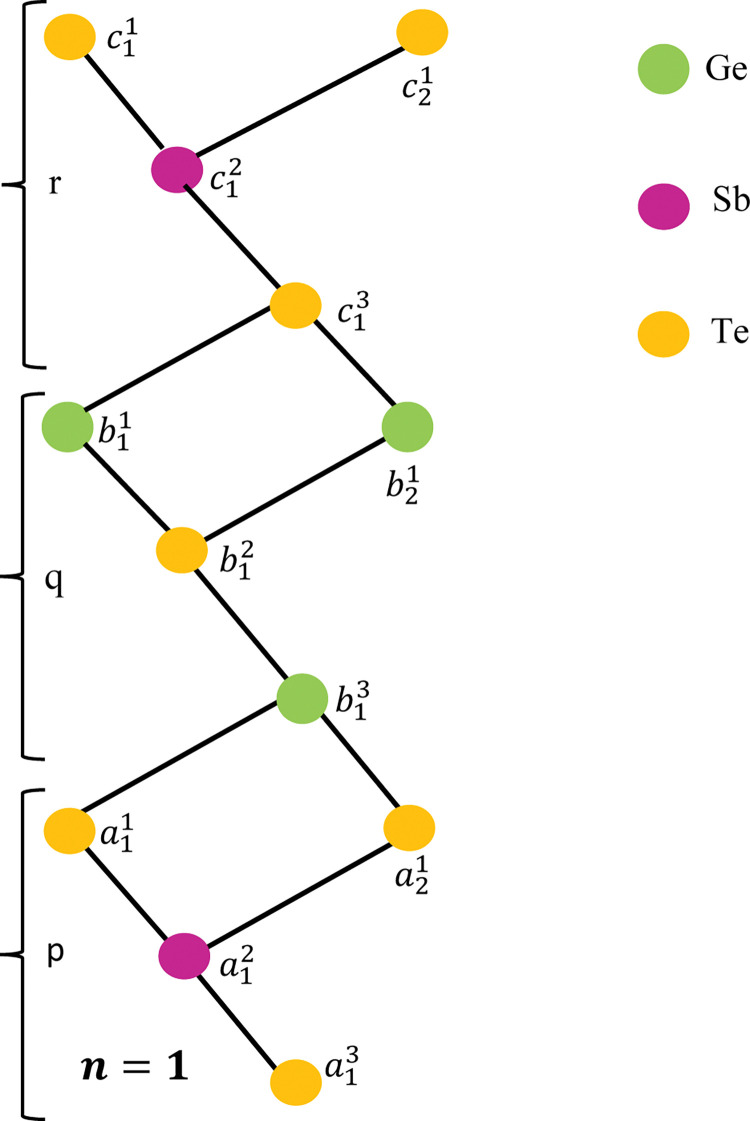
Structure of *GeSbTe* superlattice *GeSbTe*(*p*,*q*,*r)*[1].

**Fig 2:**
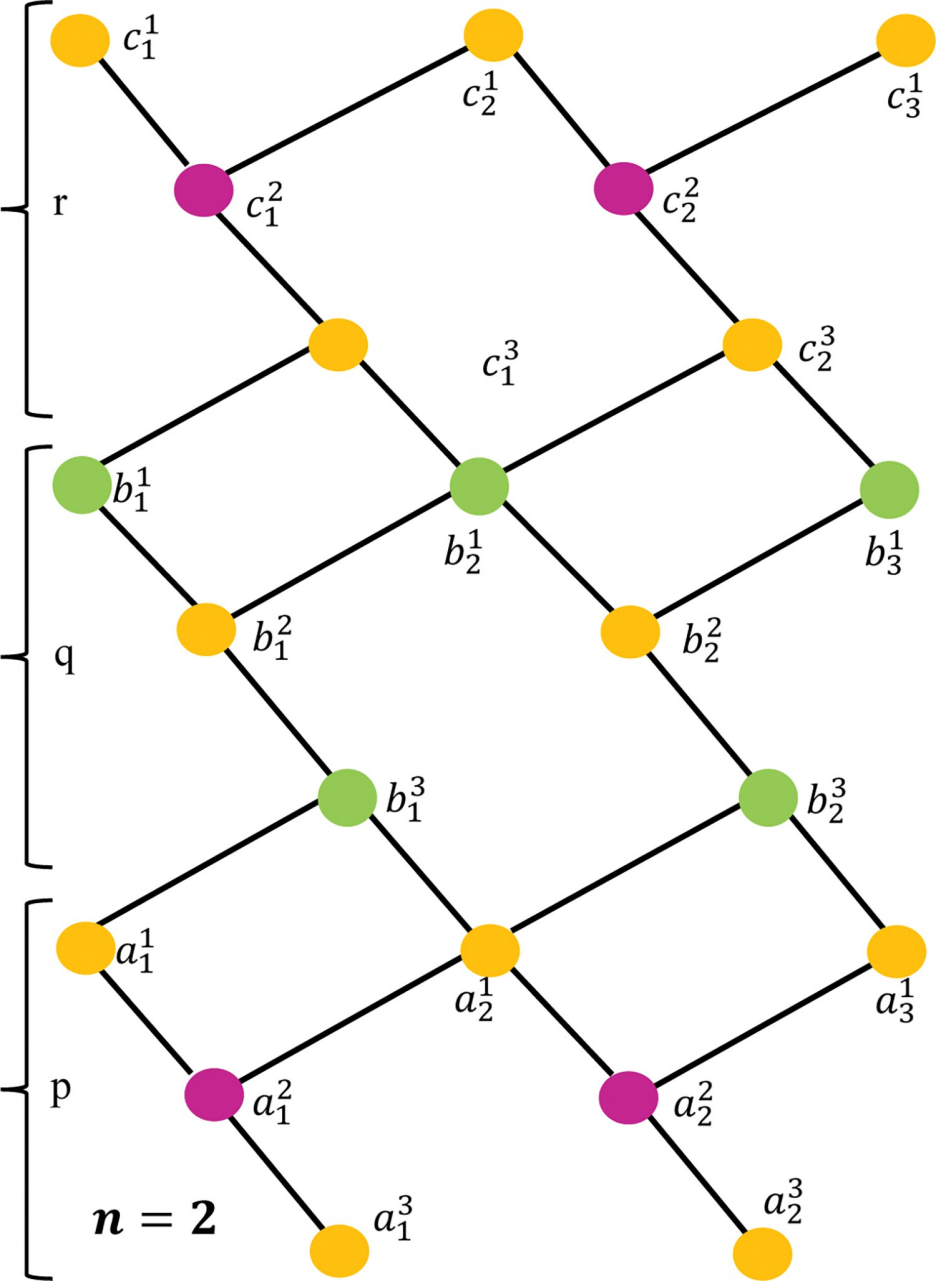
Structure of *GeSbTe* superlattice *GeSbTe*(*p*,*q*,*r)*[3].

**Fig 3:**
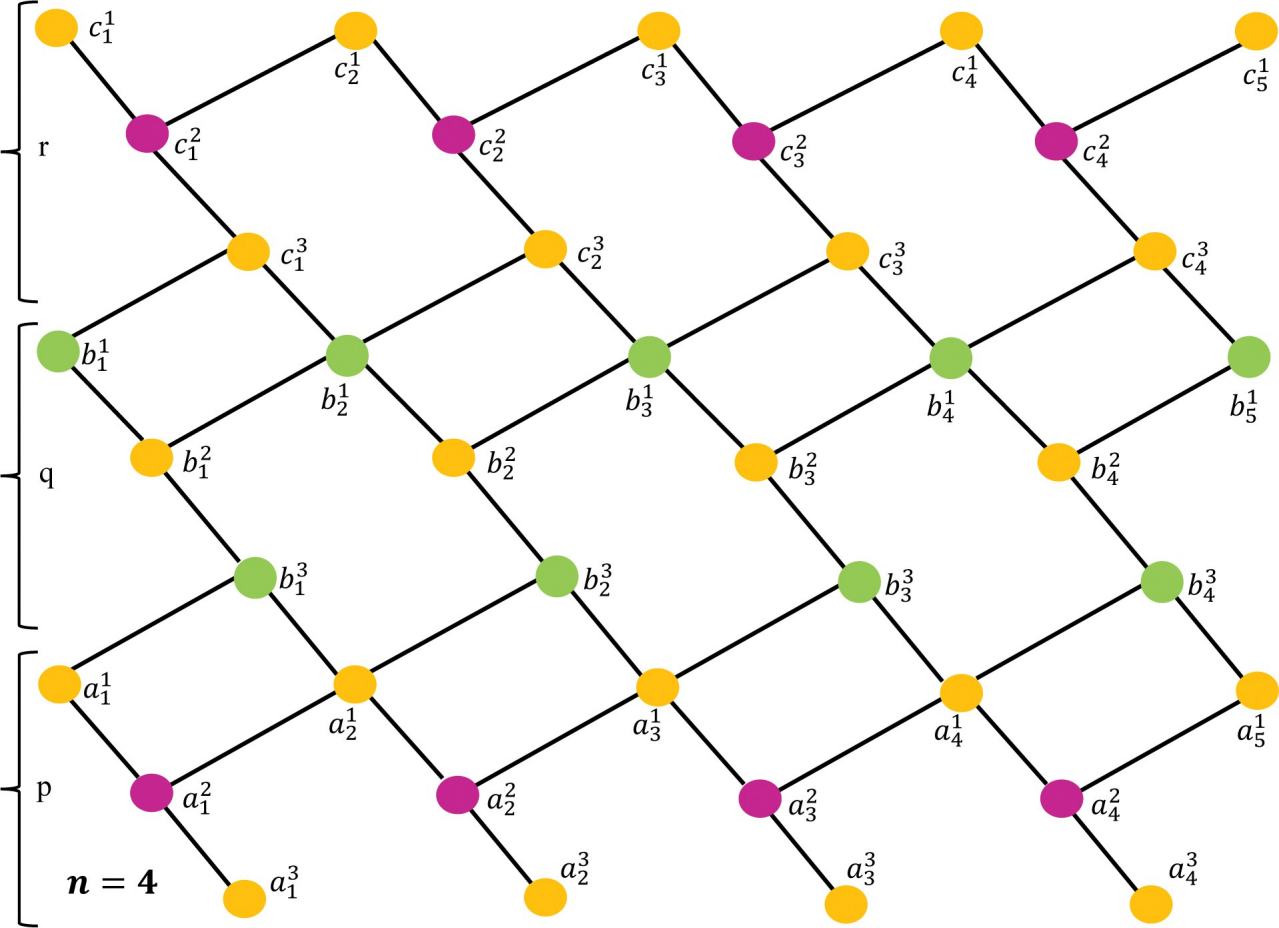
Structure of *GeSbTe* superlattice *GeSbTe*(*p*,*q*,*r)*[5].

For simplicity, we use the notation by *G*[*n*], where *n* denotes the number of unit cells of the lattice. Fig 1 depicts the unit section of *GeSbTe* superlattice where the atoms are denoted by the vertices and the edges represent the bonds between the atoms. The molecular graphs of *G*[2] and *G*[3] are shown in Figs 2 and 3 respectively. To find the resolving set of *G*[*n*], we divide the graph in to three regions namely, *p*, *q* and *r* (see Figs 1–3). Observe that each region of *G*[*n*] contains 1+3*n* vertices. In total there are 9*n*+3 vertices and 13*n* edges. The partition of *G*[*n*] based on the degree of vertices is depicted in Table 1

**Table 1:** Degree of vertices and edges for partitions of superlattice structure *GeSbTe*(*p*,*q*,*r*)[*n*].

*GeSbTe*(*p*,*q*,*r*)[*n*]	*n* = 1	*n* = 2	*n* = 4	*n* = *n*
Vertices of Degree 1	3	4	6	*n*+2
Vertices of Degree 2	4	4	4	4
Vertices of Degree 3	5	10	20	5*n*
Vertices of Degree 4	0	2	6	2*n*−2
Total Vertices	12	21	39	9*n*+3
Total Edges	13	26	52	13*n*

**Lemma # 1:** The Superlattice structure *GeSbTe*(*p*,*q*,*r*)[*n*] has a resolving set with cardinality 3.

Proof:Let $W=\{a_{1}^{1},b_{1}^{1},c_{1}^{1}\}$ is an ordered verticessubset of the GeSbTe Superlattice structure *GeSbTe*(*p*,*q*,*r*)[*n*]. We will prove that *W* is the resolving set. Given below are the representation of the vertices ofGeSbTe Superlattice structure *GeSbTe*(*p*,*q*,*r*)[*n*] with respect to *W*.


$$d(a_{i}^{p}|W)=\left\{ \begin{array}{ccc} \left(2i+p-3,\;p+2,\;p+5\right) & If\;0<i\leq 2,\; & p=1,\;2,\;3\\ \left(2i+p-3,\;p+4,\;p+5\right) & If\;i=3,\; & p=1,\;2,\;3\\ \left(2i+p-3,\;2i+p-2,\;2i+p-1\right) & If\;4\leq i\leq n,\; & p=1,\;2,\;3 \end{array}\right.)$$

$$d(b_{i}^{q}|W)=\left\{ \begin{array}{ccc} \left(-q+4,\;q-1,\;q+2\right) & If\;i=1,\; & q=1,\;2,\;3\\ \left(2i-1,\;2i+q-3,\;2i+q-2\right) & If\;i\geq 2,\; & q=1,\;3\\ \left(2i,\;2i+q-3,\;2i+q-2\right) & If\;n\geq i\geq 2,\; & q=2 \end{array}\right.$$

$$d(c_{i}^{r}|W)=\left\{ \begin{array}{ccc} \left(7-r,\;4-r,\;r-1\right) & If\;i=1, & r=1,2,3\\ \left(2i,\;2i-1,\;2i+r-3\right) & If\;i\geq 2, & r=1,3\\ \left(2i+1,\;2i,\;2i+r-3\right) & If\;n\geq i\geq 2, & r=2 \end{array}\right.$$

Since the representation of every vertex of the graph *GeSbTe*(*p*,*q*,*r*)[*n*] with the set *W* is unique, therefore the set *W* is the resolving set for the graph *GeSbTe*(*p*,*q*,*r*)[*n*].

### Special cases

Here we discuss some special cases to understand the proof of lemma 1.

**Case n = 1.** For the superlattice *GeSbTe*(*p*,*q*,*r*) [1], we have

$$d(a_{1}^{1}|W)=(0,3,6);\;d(b_{1}^{1}|W)=(3,0,3);\;d(c_{1}^{1}|W)=(6,3,0);$$

$$d(a_{1}^{2}|W)=(1,4,7);\;d(b_{1}^{2}|W)=(2,1,4);\;d(c_{1}^{2}|W)=(5,2,1);$$

$$d(a_{1}^{3}|W)=(2,5,8);\;d(b_{1}^{3}|W)=(1,2,5);\;d(c_{1}^{3}|W)=(4,1,2);$$

$$d(a_{2}^{1}|W)=(2,3,6);\;d(b_{2}^{1}|W)=(3,2,3);\;d(c_{2}^{1}|W)=(4,3,2).$$

**Case n = 2.** Similarly, for superlattice *GeSbTe*(*p*,*q*,*r*) [3], we have

$$d(a_{1}^{1}|W)=(0,3,6);\;d(b_{1}^{1}|W)=(3,0,3);\;d(c_{1}^{1}|W)=(6,3,0);$$

$$d(a_{1}^{2}|W)=(1,4,7);\;d(b_{1}^{2}|W)=(2,1,4);\;d(c_{1}^{2}|W)=(5,2,1);$$

$$d(a_{1}^{3}|W)=(2,5,8);\;d(b_{1}^{3}|W)=(1,2,5);\;d(c_{1}^{3}|W)=(4,1,2);$$

$$d(a_{2}^{1}|W)=(2,3,6);\;d(b_{2}^{1}|W)=(3,2,3);\;d(c_{2}^{1}|W)=(4,3,2);$$

$$d(a_{2}^{2}|W)=(3,4,7);\;d(b_{2}^{2}|W)=(4,3,4);\;d(c_{2}^{2}|W)=(5,4,3);$$

$$d(a_{2}^{3}|W)=(4,5,8);\;d(b_{2}^{3}|W)=(3,4,5);\;d(c_{2}^{3}|W)=(4,3,4);$$

$$d(a_{3}^{1}|W)=(4,5,6);\;d(b_{3}^{1}|W)=(5,4,5);\;d(c_{3}^{1}|W)=(6,5,4).$$

Hence $W=\{a_{1}^{1},b_{1}^{1},c_{1}^{1}\}$ is the resolving set.

**Theorem # 1:**Metric dimension of *GeSbTe* Superlattice structure *GeSbTe*(*p*,*q*,*r*)[*n*] is 3.

Proof:To prove metric dimension of *GeSbTe*(*p*,*q*,*r*)[*n*] is 3, we use the lemma 1 in which $W=\{a_{1}^{1},b_{1}^{1},c_{1}^{1}\}$ is the resolving set with cardinality 3.

We will now prove that dim(GeSbTe(p,q,r)[n])≥3. Suppose on contrary that dim(GeSbTe(p,q,r)[n]) = 2 and *W*′ is resolving set of cardinalities 2.

**Case 1:** If $W^{\prime }=\{a_{i}^{p},a_{j}^{p_{0}}\},\;(p,p_{0}\leq 3),\;(p,p_{0}\in \{1,2,3\}),\;1\leq i,1\leq j,\;i\in \{1,2,3,\ldots \ldots ,n\},\;j\in \{1,2,3,\ldots \ldots ,n\}$. Then $d(b_{l}^{q}|W\prime )=d(b_{m}^{q_{0}}|W\prime )$, where (l,m∈{1,2,…,n}), $\left(q,q_{0}\in \{1,2,3\}\right)$ and dim(GeSbTe(p,q,r)[n])≠2.

**Case 2:** If $W^{\prime }=\{b_{i}^{q},b_{j}^{q_{0}}\},\;(q,q_{0}\leq 3),\;(q,q_{0}\in \{1,2,3\}),\;1\leq i,\;1\leq j,\;i\in \{1,2,3,\ldots \ldots ,n\},\;j\in \{1,2,3,\ldots \ldots ,n\}$. Then $d(c_{l}^{r}|W\prime )=d(c_{m}^{r_{0}}|W\prime )$ where (l,m∈{1,2,…,n}), $\left(r,r_{0}\in \{1,2,3\}\right)$ and dim(GeSbTe(p,q,r)[n])≠2.

**Case 3:** If $W^{\prime }=\{c_{i}^{r},c_{j}^{r_{0}}\},\;(r,r_{0}\leq 3),\;(r,r_{0}\in \{1,2,3\}),\;1\leq i,\;1\leq j,\;i\in \{1,2,3,\ldots \ldots ,n\},\;j\in \{1,2,3,\ldots \ldots ,n\}$. Then $d(c_{l}^{r}|W\prime )=d(b_{m}^{q}|W\prime )$ where (l,m∈{1,2,…,n}), (r,q∈{1,2,3}) and dim(GeSbTe(p,q,r)[n])≠2.

**Case4:** If $W^{\prime }=\{a_{i}^{p},b_{j}^{q}\},\;\leq 3,\;q\leq 3,\;p,\;q\in \{1,2,3\},\;1\leq i,\;1\leq j,\;i\in \{1,2,3,\ldots \ldots ,n\},\;j\in \{1,2,3,\ldots \ldots ,n\}$. Then $d(a_{l}^{p}|W\prime )=d(b_{m}^{q}|W\prime )$ where (l,m∈{1,2,…,n}), (p,q∈{1,2,3}) and dim(GeSbTe(p,q,r)[n])≠2.

**Case 5:** If $W^{\prime }=\{a_{i}^{p},c_{j}^{r}\},\;p\leq 3,\;r\leq 3,\;p,\;r\in \{1,2,3\},\;1\leq i,\;1\leq j,\;i\in \{1,2,3,\ldots \ldots ,n\},\;j\in \{1,2,3,\ldots \ldots ,n\}$. Then $d(b_{l}^{q}|W\prime )=d(c_{m}^{r}|W\prime )$ where (l,m∈{1,2,…,n}), (q,r∈{1,2,3}) and dim(GeSbTe(p,q,r)[n])≠2.

**Case 6:** If $W^{\prime }=\{b_{i}^{q},c_{j}^{r}\},\;q\leq 3,\;r\leq 3,\;q,\;r\in \{1,2,3\},\;1\leq i,\;1\leq j,\;i\in \{1,2,3,\ldots \ldots ,n\},\;j\in \{1,2,3,\ldots \ldots ,n\}$. Then $d(b_{l}^{q}|W\prime )=d(b_{m}^{q_{0}}|W\prime )$ where (l,m∈{1,2,…,n}), $\left(q,q_{0}\in \{1,2,3\}\right)$ and dim(GeSbTe(p,q,r)[n])≠2.

Hence, dim(GeSbTe(p,q,r)[n])≥3.

**Lemma # 2:**TheGeSbTe Superlattice structure *GeSbTe*(*p*,*q*,*r*)[*n*] has a *fault tolerent* resolving set with cardinality 4.

Proof: To show the graph *GeSbTe*(*p*,*q*,*r*)[*n*], has *fault tolerent* resolving set with cardinality 4 and we want to prove that $W_{f}=\{a_{1}^{1},b_{1}^{1},c_{1}^{1},b_{1}^{2}\}$ is one of the *fault tolerent* resolving set.

For this we can calculate the distances of each vertex from *W*_*f*_ as follows

$$d(a_{i}^{p}|W_{f})=\left\{ \begin{array}{ccc} \left(2i+p-3,\;p+2,\;p+5,\;p+1\right) & If\;0<i\leq 2, & p=1,2,3\\ \left(2i+p-3,\;p+4,\;p+5,2i+p-3\right) & If\;i=3, & p=1,2,3\\ \left(2i+p-3,2i+p-2,2i+p-1,2i+p-3\right) & If\;n\geq i\geq 4, & p=1,2,3 \end{array}\right.$$

$$d(b_{i}^{q}|W_{f})=\left\{ \begin{array}{ccc} \begin{array}{c} \left(-q+4,\;q-1,\;q+2,\;i\right)\\ \left(-q+4,\;q-1,\;q+2,\;q-2i\right) \end{array} & \begin{array}{c} If\;i=1,\\ If\;i=1, \end{array} & \begin{array}{c} q=1,3\\ q=2 \end{array}\\ \left(2i-1,\;2i+q-3,\;2i+q-2,\;2i+q-4\right) & If\;n\geq i\geq 2, & q=1,3\\ \left(2i,\;2i+q-3,\;2i+q-2,\;2i+q-4\right) & If\;n\geq i\geq 2, & q=2 \end{array}\right.$$

$$d(c_{i}^{r}|W_{f})=\left\{ \begin{array}{ccc} \left(7-r,\;4-r,\;r-1,\;5-r\right) & If\;i=1, & r=1,2,3\\ \begin{array}{c} \left(2i,\;2i-1,\;2i+r-3,\;5-r\right)\\ \left(2i+1,2i,\;2i+r-3,\;5-r\right) \end{array} & \begin{array}{c} If\;i=2,\\ If\;i=2, \end{array} & \begin{array}{c} r=1,3\\ r=2 \end{array}\\ \begin{array}{c} \left(2i,\;2i-1,\;2i+r-3,\;2i-2\right)\\ \left(2i+1,\;2i,\;2i+r-3,\;2i-1\right) \end{array} & \begin{array}{c} If\;n\geq i\geq 3,\\ If\;n\geq i\geq 3, \end{array} & \begin{array}{c} r=1,3\\ r=2 \end{array} \end{array}\right.$$

Since the representation of every vertex of the graph *GeSbTe*(*p*,*q*,*r*)[*n*] with the set *W*_*f*_ is unique, therefore the set *W*_*f*_ is the resolving set for the graph *GeSbTe*(*p*,*q*,*r*)[*n*].

Now we want to prove that *W*_*f*_ is the fault tolerant resolving set for this we will eliminate each element one by one and show that it will again a resolving set.

If we remove $a_{1}^{1}$ from *W*_*f*_ then $W_{1}=\{b_{1}^{1},c_{1}^{1},b_{1}^{2}\}$ and

$$d(a_{i}^{p}|W_{1})=\left\{ \begin{array}{ccc} \left(p+2,\;p+5,\;p+1\right) & If\;0<i\leq 2, & p=1,2,\;3\\ \left(p+4,\;p+5,\;2i+p-3\right) & If\;i=3, & p=1,2,3\\ \left(2i+p-2,\;2i+p-1,\;2i+p-3\right) & If\;n\geq i\geq 4, & p=1,2,3 \end{array}\right.$$

$$d(b_{i}^{q}|W_{1})=\left\{ \begin{array}{ccc} \begin{array}{c} \left(q-1,\;q+2,\;i\right)\\ \left(q-1,\;q+2,\;q-2i\right) \end{array} & \begin{array}{c} If\;i=1,\\ If\;i=1, \end{array} & \begin{array}{c} q=1,3\\ q=2 \end{array}\\ \left(2i+q-3,\;2i+q-2,\;2i+q-4\right) & If\;n\geq i\geq 2, & q=1,3\\ \left(2i+q-3,\;2i+q-2,\;2i+q-4\right) & If\;n\geq i\geq 2, & q=2 \end{array}\right.$$

$$d(c_{i}^{r}|W_{1})=\left\{ \begin{array}{ccc} \left(4-r,\;r-1,\;5-r\right) & If\;i=1, & r=1,2,3\\ \begin{array}{c} \left(2i-1,\;2i+r-3,\;5-r\right)\\ \left(2i,\;2i+r-3,\;5-r\right) \end{array} & \begin{array}{c} If\;i=2,\\ If\;i=2, \end{array} & \begin{array}{c} r=1,3\\ r=2 \end{array}\\ \begin{array}{c} \left(2i-1,\;2i+r-3,\;2i-2\right)\\ \left(2i,\;2i+r-3,\;2i-1\right) \end{array} & \begin{array}{c} If\;n\geq i\geq 3,\\ If\;n\geq i\geq 3, \end{array} & \begin{array}{c} r=1,3\\ r=2 \end{array} \end{array}\right.$$

Since the representation of every vertex of the graph *GeSbTe*(*p*,*q*,*r*)[*n*] with the set *W*_1_ is unique, therefore the set *W*_1_ is the resolving set for the graph *GeSbTe*(*p*,*q*,*r*)[*n*].

If we remove $b_{1}^{1}$ from *W*_*f*_ then $W_{2}=\{a_{1}^{1},c_{1}^{1},b_{1}^{2}\}$ and

$$d(a_{i}^{p}|W_{2})=\left\{ \begin{array}{ccc} \left(2i+p-3,\;p+5,\;p+1\right) & If\;0<i\leq 2, & p=1,2,3\\ \left(2i+p-3,\;p+5,\;2i+p-3\right) & If\;i=3, & p=1,2,3\\ \left(2i+p-3,\;2i+p-1,\;2i+p-3\right) & If\;n\geq i\geq 4, & p=1,2,3 \end{array}\right.$$

$$d(b_{i}^{q}|W_{2})=\left\{ \begin{array}{ccc} \begin{array}{c} \left(-q+4,\;q+2,\;i\right)\\ \left(-q+4,\;q+2,\;q-2i\right) \end{array} & \begin{array}{c} If\;i=1,\\ If\;i=1, \end{array} & \begin{array}{c} q=1,3\\ q=2 \end{array}\\ \left(2i-1,\;2i+q-2,\;2i+q-4\right) & If\;n\geq i\geq 2, & q=1,3\\ \left(2i,\;2i+q-2,\;2i+q-4\right) & If\;n\geq i\geq 2, & q=2 \end{array}\right.$$

$$d(c_{i}^{r}|W_{2})=\left\{ \begin{array}{ccc} \left(7-r,\;r-1,\;5-r\right) & If\;i=1, & r=1,2,3\\ \begin{array}{c} \left(2i,\;2i+r-3,\;5-r\right)\\ \left(2i+1,\;2i+r-3,\;5-r\right) \end{array} & \begin{array}{c} If\;i=2,\\ If\;i=2, \end{array} & \begin{array}{c} r=1,3\\ r=2 \end{array}\\ \begin{array}{c} \left(2i,\;2i+r-3,\;2i-2\right)\\ \left(2i+1,\;2i+r-3,\;2i-1\right) \end{array} & \begin{array}{c} If\;n\geq i\geq 3,\\ If\;n\geq i\geq 3, \end{array} & \begin{array}{c} r=1,3\\ r=2 \end{array} \end{array}\right.$$

Since the representation of every vertex of the graph *GeSbTe*(*p*,*q*,*r*)[*n*] with the set *W*_2_ is unique, therefore the set *W*_2_ is the resolving set for the graph *GeSbTe*(*p*,*q*,*r*)[*n*].

If we remove $c_{1}^{1}$ from *W*_*f*_ then $W_{2}=\{a_{1}^{1},b_{1}^{2},b_{1}^{1}\}$ and

$$d(a_{i}^{p}|W_{3})=\left\{ \begin{array}{ccc} \left(2i+p-3,\;p+2,\;p+1\right) & If\;0<i\leq 2, & p=1,2,3\\ \left(2i+p-3,\;p+4,\;2i+p-3\right) & If\;i=3, & p=1,2,3\\ \left(2i+p-3,2i+p-2,\;2i+p-3\right) & If\;n\geq i\geq 4, & p=1,2,3 \end{array}\right.$$

$$d(b_{i}^{q}|W_{3})=\left\{ \begin{array}{ccc} \begin{array}{c} \left(-q+4,\;q-1,\;i\right)\\ \left(-q+4,\;q-1,\;q-2i\right) \end{array} & \begin{array}{c} If\;i=1,\\ If\;i=1, \end{array} & \begin{array}{c} q=1,3\\ q=2 \end{array}\\ \left(2i-1,\;2i+q-3,\;2i+q-4\right) & If\;n\geq i\geq 2, & q=1,3\\ \left(2i,\;2i+q-3,\;2i+q-4\right) & If\;n\geq i\geq 2, & q=2 \end{array}\right.$$

$$d(c_{i}^{r}|W_{3})=\left\{ \begin{array}{ccc} \left(7-r,\;4-r,\;5-r\right) & If\;i=1, & r=1,2,3\\ \begin{array}{c} \left(2i,\;2i-1,\;5-r\right)\\ \left(2i+1,\;2i,\;5-r\right) \end{array} & \begin{array}{c} If\;i=2,\\ If\;i=2, \end{array} & \begin{array}{c} r=1,3\\ r=2 \end{array}\\ \begin{array}{c} \left(2i,\;2i-1,\;2i-2\right)\\ \left(2i+1,\;2i,\;2i-1\right) \end{array} & \begin{array}{c} If\;i\geq 3,\\ If\;i\geq 3, \end{array} & \begin{array}{c} r=1,3\\ r=2 \end{array} \end{array}\right.$$

Since the representation of every vertex of the graph *GeSbTe*(*p*,*q*,*r*)[*n*] with the set *W*_3_ is unique, therefore the set *W*_3_ is the resolving set for the graph *GeSbTe*(*p*,*q*,*r*)[*n*].

If we remove $b_{1}^{2}$ from *W*_*f*_ then it becomes again $W=\{a_{1}^{1},b_{1}^{1},c_{1}^{1}\}$

$$d(a_{i}^{p}|W)=\left\{ \begin{array}{ccc} \left(2i+p-3,\;p+2,\;p+5\right) & If\;0<i\leq 2, & p=1,2,3\\ \left(2i+p-3,\;p+4,\;p+5\right) & If\;i=3, & p=1,2,3\\ \left(2i+p-3,\;2i+p-2,\;2i+p-1\right) & If\;n\geq i\geq 4, & p=1,2,3 \end{array}\right.$$

$$d(b_{i}^{q}|W)=\left\{ \begin{array}{ccc} \left(-q+4,\;q-1,\;q+2\right) & If\;i=1, & q=1,2,3\\ \left(2i-1,\;2i+q-3,\;2i+q-2\right) & If\;n\geq i\geq 2, & q=1,3\\ \left(2i,\;2i+q-3,\;2i+q-2\right) & If\;n\geq i\geq 2, & q=2 \end{array}\right.$$

$$d(c_{i}^{r}|W)=\left\{ \begin{array}{ccc} \left(7-r,\;4-r,\;\;r-1\right) & If\;i=1, & r=1,2,3\\ \left(2i,\;2i-1,\;2i+r-3\right) & If\;n\geq i\geq 2, & r=1,3\\ \left(2i+1,\;2i,\;2i+r-3\right) & If\;n\geq i\geq 2, & r=2 \end{array}\right.$$

Since the representation of every vertex of the graph *GeSbTe*(*p*,*q*,*r*)[*n*] with the set *W*_4_ is unique, therefore the set *W*_4_ is the resolving set for the graph *GeSbTe*(*p*,*q*,*r*)[*n*].

Hence proved that *W*_*f*_ is the fault resolving set.

### Special case n = 1

Here we discuss a special case n = 1 to understand the proof of lemma 2.

The set $W_{f}=\{a_{1}^{1},b_{1}^{1},c_{1}^{1},b_{1}^{2}\}$ is the fault resolving set.By using the results of lemma 2 for *n* = 1, we have

$$d(a_{1}^{1}|W_{f})=(0,3,6,2);\;d(b_{1}^{1}|W_{f})=(3,0,3,1);\;d(c_{1}^{1}|W_{f})=(6,3,0,4);$$

$$d(a_{1}^{2}|W_{f})=(1,4,7,3);\;d(b_{1}^{2}|W_{f})=(2,1,4,0);\;d(c_{1}^{2}|W_{f})=(5,2,1,3);$$

$$d(a_{1}^{3}|W_{f})=(2,5,8,4);\;d(b_{1}^{3}|W_{f})=(1,2,5,1);\;d(c_{1}^{3}|W_{f})=(4,1,2,3);$$

$$d(a_{2}^{1}|W_{f})=(2,3,6,2);\;d(b_{2}^{1}|W_{f})=(3,2,3,1);\;d(c_{2}^{1}|W_{f})=(4,3,2,4);$$

If we remove $a_{1}^{1}$ from *W*_*f*_ then $W_{1}=\{b_{1}^{1},c_{1}^{1},b_{1}^{2}\}$. By using the results of lemma 2 for *n* = 1, we have

$$d(a_{1}^{1}|W_{1})=(3,6,2);\;d(b_{1}^{1}|W_{1})=(0,3,1);\;d(c_{1}^{1}|W_{1})=(3,0,4);$$

$$d(a_{1}^{2}|W_{1})=(4,7,3);\;d(b_{1}^{2}|W_{1})=(1,4,0);\;d(c_{1}^{2}|W_{1})=(2,1,3);$$

$$d(a_{1}^{3}|W_{1})=(5,8,4);\;d(b_{1}^{3}|W_{1})=(2,5,1);\;d(c_{1}^{3}|W_{1})=(1,2,3);$$

$$d(a_{2}^{1}|W_{1})=(3,6,2);\;d(b_{2}^{1}|W_{1})=(2,3,1);\;d(c_{2}^{1}|W_{1})=(3,2,4);$$

If we remove $b_{1}^{1}$ from *W*_*f*_ then $W_{2}=\{a_{1}^{1},c_{1}^{1},b_{1}^{2}\}$. By using the results of lemma 2 for *n* = 1, we have

$$d(a_{1}^{1}|W_{2})=(0,6,2);\;d(b_{1}^{1}|W_{2})=(3,3,1);\;d(c_{1}^{1}|W_{2})=(6,0,4);$$

$$d(a_{1}^{2}|W_{2})=(1,7,3);\;d(b_{1}^{2}|W_{2})=(2,4,0);\;d(c_{1}^{2}|W_{2})=(5,1,3);$$

$$d(a_{1}^{3}|W_{2})=(2,8,4);\;d(b_{1}^{3}|W_{2})=(1,5,1);\;d(c_{1}^{3}|W_{2})=(4,2,3);$$

$$d(a_{2}^{1}|W_{2})=(2,6,2);\;d(b_{2}^{1}|W_{2})=(3,3,1);\;d(c_{2}^{1}|W_{2})=(4,2,4);$$

If we remove $c_{1}^{1}$ from *W*_*f*_ then $W_{3}=\{a_{1}^{1},b_{1}^{1},b_{1}^{2}\}$. By using the results of lemma 2 for *n* = 1, we have

$$d(a_{1}^{1}|W_{3})=(0,3,2);\;d(b_{1}^{1}|W_{3})=(3,0,1);\;d(c_{1}^{1}|W_{3})=(6,3,4);$$

$$d(a_{1}^{2}|W_{3})=(1,4,3);\;d(b_{1}^{2}|W_{3})=(2,1,0);\;d(c_{1}^{2}|W_{3})=(5,2,3);$$

$$d(a_{1}^{3}|W_{3})=(2,5,4);\;d(b_{1}^{3}|W_{3})=(1,2,1);\;d(c_{1}^{3}|W_{3})=(4,1,3);$$

$$d(a_{2}^{1}|W_{3})=(2,3,2);\;d(b_{2}^{1}|W_{3})=(3,2,1);\;d(c_{2}^{1}|W_{3})=(4,3,4);$$

If we remove $b_{1}^{2}$ from *W*_*f*_ then it becomes again $W=\{a_{1}^{1},b_{1}^{1},c_{1}^{1}\}$. By using the results of lemma 2 for *n* = 1, we have

$$d(a_{1}^{1}|W)=(0,3,6);\;d(b_{1}^{1}|W)=(3,0,3);\;d(c_{1}^{1}|W)=(6,3,0);$$

$$d(a_{1}^{2}|W)=(1,4,7);\;d(b_{1}^{2}|W)=(2,1,4);\;d(c_{1}^{2}|W)=(5,2,1);$$

$$d(a_{1}^{3}|W)=(2,5,8);\;d(b_{1}^{3}|W)=(1,2,5);\;d(c_{1}^{3}|W)=(4,1,2);$$

$$d(a_{2}^{1}|W)=(2,3,6);\;d(b_{2}^{1}|W)=(3,2,3);\;d(c_{2}^{1}|W)=(4,3,2);$$

Hence proved that *W*_*f*_ is the fault resolving set.

**Theorem # 2:**The fault tolerant metric dimension of GeSbTe Superlattice structure *GeSbTe*(*p*,*q*,*r*)[*n*] is 4.

From lemma 2 we see that the cardinality of *W*_*f*_ is 4 and from Theorem 1, we proved that the metric dimension of *GeSbTe*(*p*,*q*,*r*)[*n*] is 3, hence the fault tolerant metric dimension of *GeSbTe*(*p*,*q*,*r*)[*n*] is 4.

## Conclusion

Metric dimension is a concept in graph theory that measures how effectively a set of vertices in a graph can be used to uniquely identify other vertices. It has applications in many fields including technology, Sciences and Social Sciences. In particular it is useful in location determination problems, where the goal is to find the position of an object or event based on limited measurements or observations. By selecting a minimal set of nodes with high metric dimension, we can accurately determine the location of the target object or event. In this work, we have computed the metric dimension of *GeSbTe* superlattice. The obtained results may be useful for better understanding the structure. The metric dimension of other material that is useful in the field of phase change memory can be calculated in future.
